# Supplementary material for: Exosomes Derived from Adipose Mesenchymal Stem Cells Promote Diabetic Chronic Wound Healing through SIRT3/SOD2
Source: Cells. 2022 Aug 18;11(16):2568. doi: 10.3390/cells11162568 (PMC9406299; doi:10.3390/cells11162568)

Table S1 Specify acronyms in figures.

ADSC-exos	Adipose mesenchymal stem cell-derived exosomes
HUVECs	Human Umbilical Vein Endothelial Cells
ROS	Reactive Oxygen Species
SIRT3	Sirtuin 3
SOD2	Superoxide Dismutase 2
EPC	Endothelial Progenitor Cell
MMP	Mitochondrial Membrane Potential
FBG	Fasting Blood Glucose
MDA	Malondialdehyde
T-AOC	Total Antioxidant Capacity
SOD	Superoxide Dismutase
CCK-8	cell counting kit-8
AC-SOD2	Acetylated Superoxide Dismutase 2
ANG1	Angiopoietin-1
FLK1	fetal liver kinase-1
VASH1	Vasohibin 1
TSP1	thrombospondin-1

Figure S1 blood vessel density in db/db mice wound.

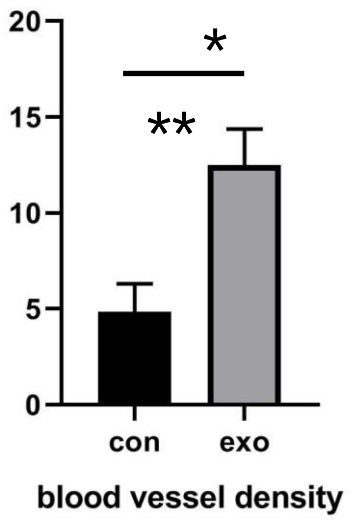

Supplement: Supplementary file 1 [file cells-11-02568-s001.zip › cells-1833129-supplementary.pdf]
